# Isolated Clones of a Human Colorectal Carcinoma Cell Line Display Variation in Radiosensitivity Following Gamma Irradiation

**DOI:** 10.1177/15593258221113797

**Published:** 2022-09-10

**Authors:** Rhea Desai, Colin Seymour, Carmel Mothersill

**Affiliations:** 1 Department of Biology, 3710 McMaster University, Hamilton, ON, Canada

**Keywords:** radiation-induced bystander effects, radiation, radiosensitivity, ionizing radiation, genetic instability, clonogenic assay

## Abstract

**Objective:**

To determine whether the width of the shoulder and the size of the bystander effect are correlated using clonal lineages derived from a cultured cell line.

**Methods:**

HCT 116 (p53 wildtype) cells were grown at cloning density and individual viable colonies were picked off and grown to establish a series of cell lines from both unirradiated and irradiated progenitors. These cell lines were then irradiated to generate full survival curves. Highly variant clones were then tested to determine the level of the bystander effect using a medium transfer protocol.

**Results:**

The multi-target model gave the best fit in these experiments and size of the shoulder *n* is assessed in terms of radiosensitivity. The parent cell line has an *n* value of 1.1 while the most variant clones have *n* values of 0.88 (Clone G) and 5.5 (Clone A). Clonal lines subject to irradiation prior to isolation differed in bystander signal strength in comparison to clonal lines which were not initially irradiated (*P* = .055).

**Conclusions:**

Based on these experiments we suggest there may be a link between shoulder size of a mammalian cell line and the strength of a bystander effect produced in vitro. This may have implications for radiotherapy related to out-of-field effects.

## Introduction

The impacts of ionizing radiation (IR) on human cells are important for radiation protection, environmental risk assessment, and radiation therapy.^1^ Recently, the effects of low dose IR have gained attention due both to the increasing use of IR in medical diagnostics, the use of novel protocols in radiotherapy such as FLASH and MRT, and the interest in small modular reactors as energy sources in remote environments.^1,2^ High dose direct IR generally leads to significant cell death through processes such as reproductive death or apoptosis^3-5^ while non-targeted and low dose radiation appears to involve other mechanisms.^1,2,6,7^ Radiation-induced bystander effects (RIBE) are of particular interest since they involve cell killing, transformation and initiation of cell signaling pathways in cells that have not been directly exposed to IR but have received signals from directly exposed cells.^2,8-14^ RIBE have been widely studied both in vivo and in vitro and they appear to be associated with low dose radiosensitivity^1,9,10,12,13,15-18^ with some suggestion that they require wildtype p53 to be expressed.^19^ This is relevant since many tumors have compromised p53 function,^20-22^ meaning that additional killing due to RIBE would predominantly affect normal cells around the tumor rather than the tumor itself. However, the research in this area is quite controversial with contradictory reports about RIBE even in laboratories using the same protocols and cells.^23^ A possible explanation for this is “drift” within cultured cell lines leading to clonal heterogeneity in populations of genetically identical cells. To test whether this might be a factor, we decided to revisit clonal heterogeneity with respect to clonal sensitivity. Through investigation of clonal populations we aim to approach in a more systematic way the often heterogenous nature of malignancies.^24,25^ The literature often refers to clonal heterogeneity within a tumor as a “fuel for resistance” and studying this key challenge in optimizing individual therapies is necessary to advance cancer treatment.^24,25^ Since radiotherapy can lead to second malignancies,^26-28^ some cell lines were derived from cultures of cells exposed to 1 Gy to determine whether there was greater variability in terms of radiosensitivity in these lines. With a better understanding of the heterogeneity of response in clonal sub-populations we may gain a new perspective which could improve radiation treatment.^14^

## Methods

### Human Cell Cultures

The immortalized human epithelial HCT116 (p53 wildtype) cell line derived from a large intestine/colon carcinoma was used in this study. Clonal cell lines were isolated from this parent cell line. These cells were routinely cultured in Roswell Park Memorial Institute (RPMI) 1640 growth medium supplemented with 10% fetal bovine serum (FBS), 100 U/mL penicillin, 100 ug/mL streptomycin, and 2.05 mM L-Glutamine. This growth medium was also used in the bystander effect assays. Cells were grown in 75 cm^2^ Falcon tissue culture flasks at 37°C and 5% CO_2_. Subcultures were conducted using 0.25% phenol red-free trypsin solution with 0.192 mM EDTA every 6-7 days. Trypsinized cells were neutralized using a greater volume of growth media. Cell cultures were 70–80% confluent upon culture. Cell concentrations were determined using Bio-Rad TC20 automated cell counter (Bio-Rad Life Science Research Divison, Canada). All reagents were purchased from Gibco, ThermoFisher Scientific.

### Clonal Isolation

**Figure 1.**
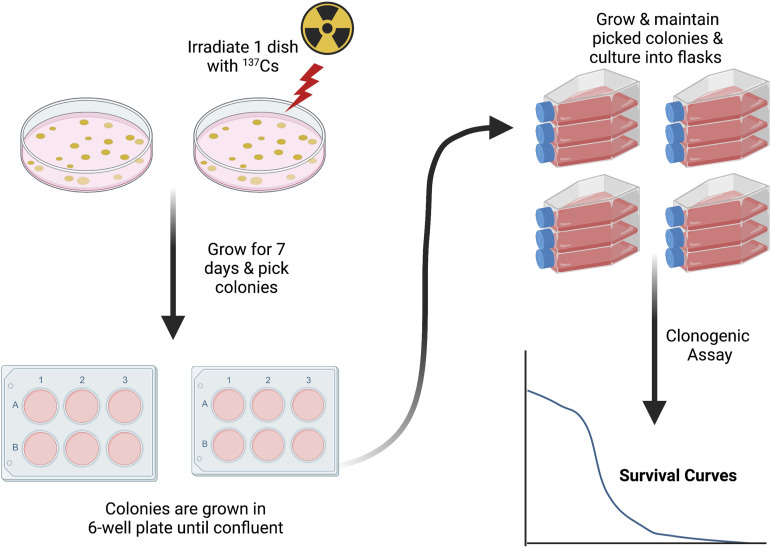
Diagram of clonal isolation methods.

Petri dishes were seeded with 200–300 cells. These were allowed to form viable colonies of at least 50 cells. After 7 days, individual clones of various sizes were chosen for clonal expansion. These colonies were scraped off the dish and resuspended in small multiwall plates (Falcon, 6-Well Flat Bottom Tissue Culture Plate, VWR Canada). The clones were passaged into T25 flasks when confluent and grown to produce a sufficient supply of cells for the experiments. Some clones were isolated from plates where cells had been exposed to 1 Gy radiation after cells had adhered to the culture plate in order to examine the effect of this dose on subsequent clonal heterogeneity (Figure 1).

### Irradiation

All irradiations were performed using a Cesium-137 gamma-emitting source with a dose rate of 198.4 mGy/min and flasks were placed 30 cm away from the source (Taylor Radiobiology Source, McMaster University). Direct irradiations for survival curve data generation were conducted 15–20 hours post seeding. Irradiations to generate medium for bystander effect assays were also completed in this manner.

### Clonogenic Survival Assay and Survival Curves

Flasks containing approximately 70% confluent cultures were used for clonogenic survival assays. Cells were removed from the flask using a Trypsin-EDTA working solution described above. Detached cells were neutralized with growth medium and mixed to form a single cell suspension. These cells were counted and plated to perform a clonogenic assay using the method described by Puck and Marcus.^29^ Cell seeding densities were determined using the plating efficiencies (PE) determined for each clonal cell line. Clonogenic assays were conducted to develop full survival curves upon irradiation of cells at the following dose points: 0, 0.5, 1.0, 3.0, 5.0, 7.0, 10.0, and 15.0 Gy. This wide range in dose points provided an overall assessment at cell survival across doses. Flasks were irradiated at the appropriate dose and returned to the incubator immediately following irradiation and grown for nine days at 37°C in an atmosphere of 5% CO_2_ in air. On day nine, all flasks were stained with 15% Carbol Fuchsin solution (Ziehl Neelson, Millipore Sigma). Colonies were counted manually to determine the surviving fraction for each dose. The data were entered into GraphPad Prism 8 software (GraphPad Software Inc., LaJolla, CA), to generate survival curve graphs.

### Bystander Effect Assay

**Figure 2.**
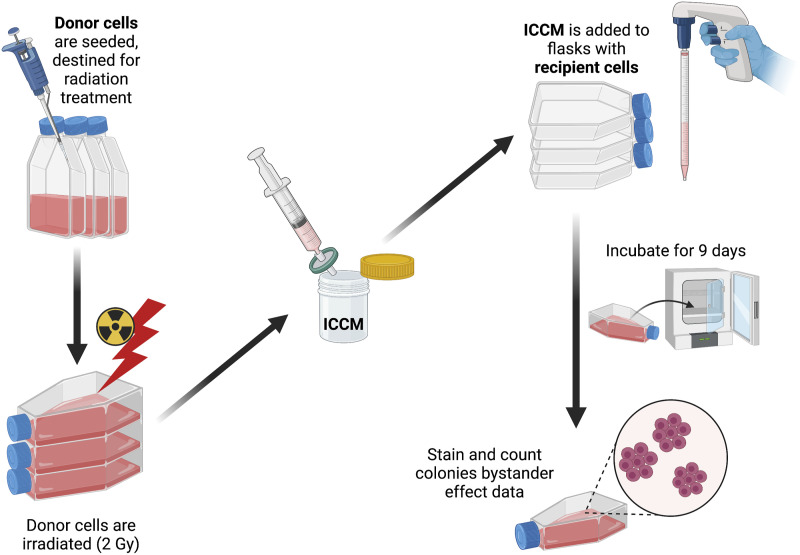
Diagram of bystander effect assay methods.

Falcon tissue culture flasks (25 cm^2^) were seeded with cells in 5 mL growth media for the following treatments and incubated for 6 hours at 37°C and 5% CO_2_: plating efficiency, direct irradiation (2.0 Gy), bystander effect donor and recipient, and sham donor and recipient (Figure 2). Sham donor flasks were not irradiated but a medium transfer was completed from sham donor flasks to sham recipient flasks to ensure there was not an effect of medium change. All donor flasks were seeded with 100 000 cells while all other flasks were seeded with 200 cells. After 6 hours, the direct irradiation and bystander effect donor flasks were irradiated with the Cesium-137 source at 2 Gy. Following irradiation, flasks were immediately returned to the incubator for 1 hour. After 1 hour of incubation, medium transfer of donor flasks was completed. The medium from donor flasks was filtered using a 0.22-μm filter and 30 mL plastic syringe (Millipore Sigma) to ensure no cells were present in the irradiated cell culture medium (ICCM). Approximately ∼15 mL media was collected from triplicate donor flasks. Growth medium from the recipient flasks was then poured off as waste and the previously filtered ICCM was added to the recipient flasks. This method of medium transfer was used for both the bystander effect and sham treatment flasks. All flasks were grown for 9 days and then stained with 15% Carbol Fuschin and counted manually.

### Statistical Analysis

For all survival curves, data are presented as a mean of three replicates in three independent trials (n=9). Least square error linear regression analyses were performed on data to produce the multi-target and linear-quadratic models using GraphPad Prism 8. Data for bystander effect assays were also collected as mean of three replicates in three independent trials. Standard error of the mean error bars are used in all figures. To determine variance between groups in bystander effect experiments, t-tests were conducted between the sham and bystander groups and the sham and direct irradiation groups for each cell line. These post hoc analyses were performed using Welch’s t-test. Significance was determined at the 95% confidence interval.

## Results

### Survival Curves

**Figure 3.**
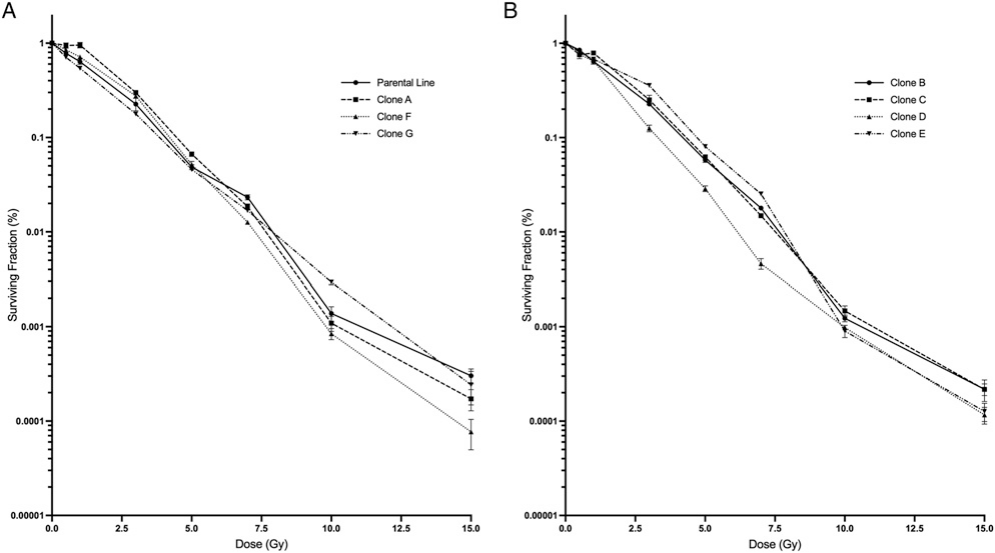
Survival curves of parental line, non-irradiated and irradiated progeny cell lines. (A) Parental and non- irradiated cell lines (parental, clone A, clone F, and clone G). (B) Irradiated clones (clones B, C, D, and E). Error bars are SEM for n = 9.

Figure 3 displays the parent HCT116 p53^+/+^ line alongside all clonal cell lines derived from either an irradiated or non-irradiated population prior to clone isolation. Data were not fitted to any established model for this figure. Clonogenic survival over a dose range of 0–15 Gy after direct exposure to a cesium-137 gamma source shows variation between each clonal line. Plating efficiency variations were also observed between clonal lines and are presented in Table 1.

**Table 1.** Summary of survival curve parameters obtained through survival curve fitting with the linear-quadratic and multitarget models for radiation-induced cell killing. Values *n* and D_0_ determined using the multi-target model. Alpha and beta values determined using the linear-quadratic model.


A. Cell lines derived from a control population HCT 116 p53+/+ population								
Parameter	Parent Line	CI (95%)	Clone A	CI (95%)	Clone F	CI (95%)	Clone G	CI (95%)
Multi-target								
*n*	1.1	0.92–1.4	5.5	3.1–30	1.5	1.3–1.7	0.88	0.82–0.93
D_0_	1.8	1.5–2.2	1.1	0.68–1.4	1.8	1.6–2.0	1.8	1.7–2.0
r^2^	0.96		0.95		0.98		1.0	
Linear-quadratic								
α	0.44	0.36–0.53	−0.058	−0.16 to 0.042	0.28	0.23–0.34	0.64	0.61–0.67
β	0.021	−0.0056 to 0.057	0.15	0.10–0.20	0.052	0.033–0.074	−0.013	−0.021 to 0.0030
r^2^	0.97		0.95		0.98		1.0	
Plating efficiency (%)	34		32		29		26	
B. Cell lines derived from a previously 1 Gy irradiated HCT 116 p53+/+ population								
Parameter	Clone B	CI (95%)	Clone C	CI (95%)	Clone D	CI (95%)	Clone E	CI (95%)
Multi-target								
*n*	1.3	1.1–1.6	1.4	1.0–1.8	1.4	1.2–1.7	1.1	0.96–1.2
D_0_	1.7	1.4–2.0	1.8	1.5–2.3	1.4	1.2–1.6	2.3	2.1–2.6
r^2^	0.97		0.94		0.98		0.98	
Linear-quadratic								
α	0.38	0.29–0.45	0.2847	0.19–0.38	0.4006	0.33–0.47	0.3441	0.30–0.39
β	0.040	0.010–0.078	0.053	0.018–0.096	0.085	0.047–0.13	0.018	0.0060–0.031
r^2^	0.97		0.95		0.98		0.98	
Plating efficiency (%)	34		33		24		27	

Note. *D*_*0*_ multiple event cell killing, *n* size of curve shoulder, α cell killing proportional to dose, β cell killing proportional to square of the dose, CI (95%) confidence interval.

**Figure 4.**
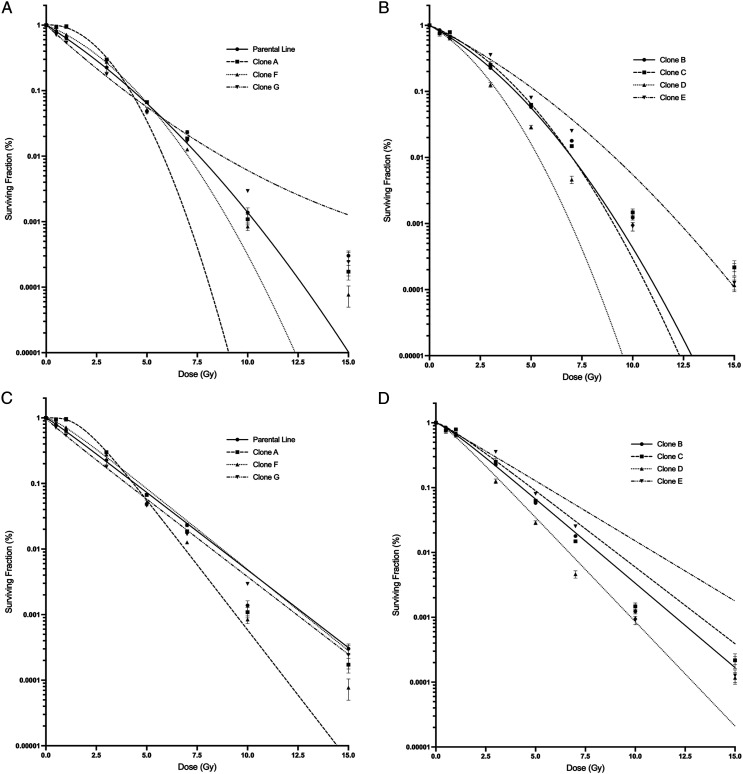
Survival curves of parental line, non-irradiated and irradiated progeny cell lines fit with either the multi- target or linear-quadratic model. The parental line and clones A, F, and G are derived from an initial population not exposed to radiation. Clones B, C, D, and E were initially treated with 1.0 Gy prior to clone isolation. (A) Parental line and clones A, F, and G fitted with the linear-quadratic model. (B) Clones B, C, D, and E fitted with the linear-quadratic model. (C) Parental line and clones A, F, and G fitted with the multitarget model. (D) Clones B, C, D, and E fitted with the multi-target model.

The linear-quadratic and multi-target models were both fitted to all survival curve data. Using the linear-quadratic model, parameters alpha and beta were noted to demonstrate variation in radiosensitivity (Table 1). Here, we see unexpected negative values for alpha of clone A and beta for clone G (Table 1A). Alpha and beta values indicate cell killing proportional to the dose and cell killing proportional to the square of the dose, respectively. Negative values for such parameters have no physical meaning and suggest the fitted equation is inadequate to describe the present data. Using the multi-target model, parameters *n* and D_0_ are observed where *n* is a common indicator for low dose radiosensitivity or the size of the cell survival curve shoulder. In comparison to the parent cell line, Clone A had the largest *n* value of 5.5 while Clone G had the smallest *n* value of 0.88 (Table 1A). A prominent shoulder for Clone A can be seen in Figure 4A and C. Both clone A and clone G were derived from unirradiated progenitor cells. It is apparent that curve fitting parameters obtained through either model show variation in radiosensitivity indicating the presence of heterogeneity in the initial cell HCT116 p53^+/+^ cell population. These curve fitting models highlight differences in cell survival response to consistent radiation doses; however, the multi-target model provided an overall better fit to data presented in this study.

### Bystander Effects

**Figure 5.**
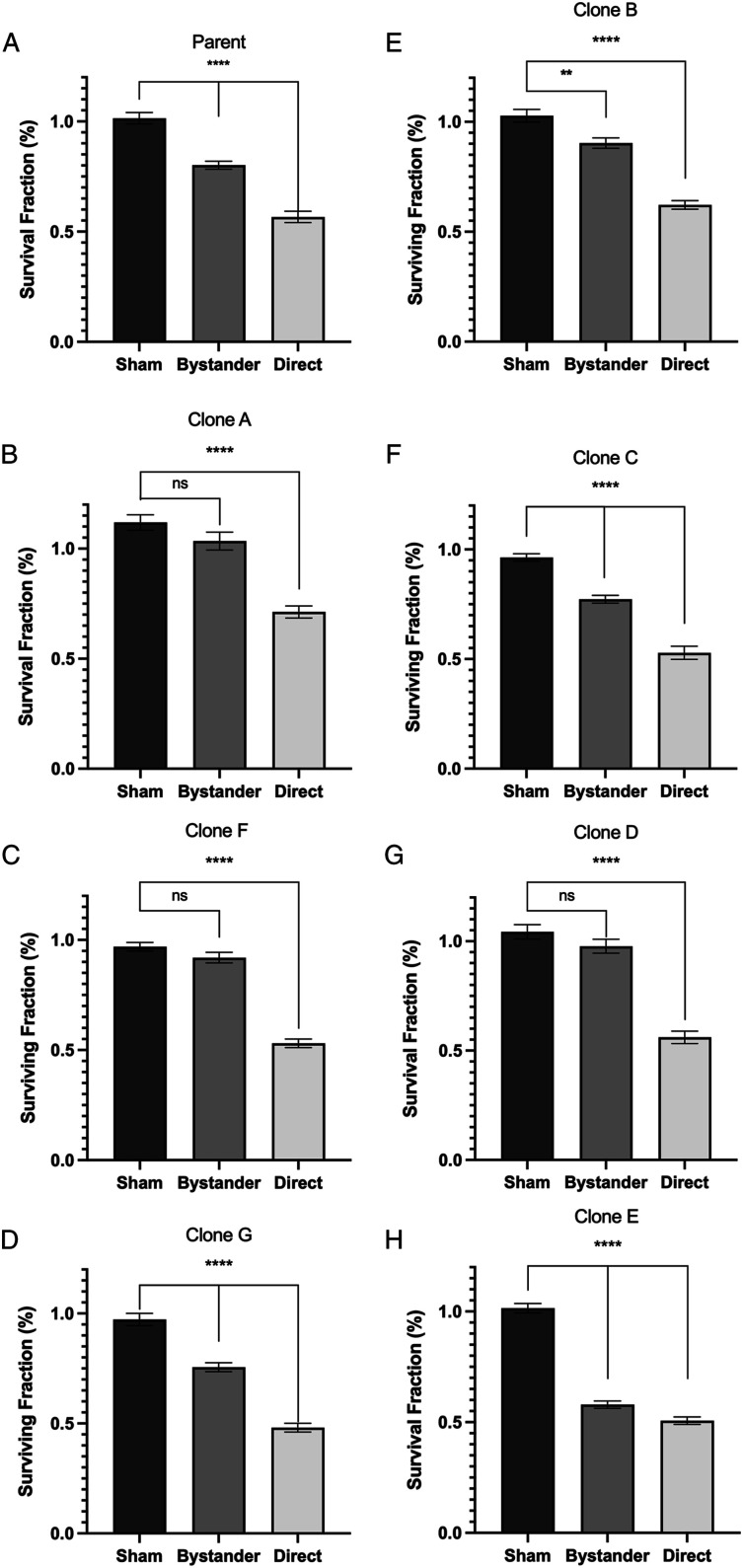
Recipient cells exposed to culture medium from irradiated cells. Sham represents cells exposed to cell culture control medium. Bystander represents cells exposed to irradiated cell culture medium collected from donor cells irradiated with 2.0 Gy. Direct represents cells irradiated directly with 2.0 Gy. (A-D) Bystander effect assay conducted on parent cell line and isolated clones not subject to irradiation prior to isolation. (E–H) Bystander effect assay conduced on isolated clones subject to irradiation prior to isolation. In all bystander effect assays, both recipient and donor cells are of the same parent or clonal line. All data is presented as the mean ± SEM (n = 9). (****P < .0001), (**P < .005) indicates a significant difference between treatment groups and sham.

Bystander effect assays were conducted to investigate differences in bystander signal strength in the clonal cell lines. Figure 5 displays a response in each clonal line following a bystander medium transfer treatment. In each bystander assay donor and reporter cells are of the same clonal line so that irradiated cell culture medium (ICCM) is filtered from donor cells of a clonal line and added to reporter cells of the same clonal line. Direct groups in Figure 5 for all parent and clonal lines were exposed to 2 Gy direct gamma irradiation and subsequently, an expected significant decrease in cell survival compared to the sham group is observed.

**Figure 6.**
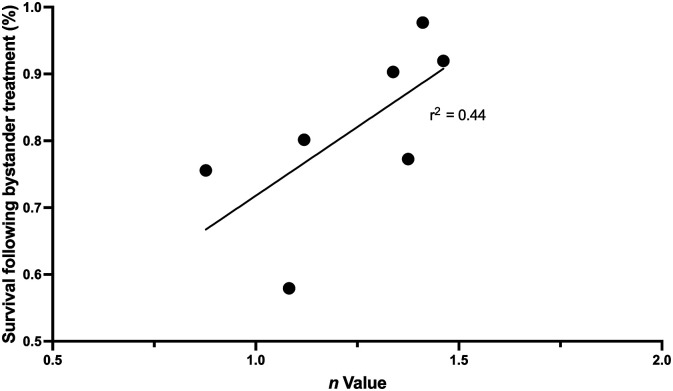
Correlation plot between n value or size of survival curve shoulder and percent cell survival following radiation-induced bystander treatment. Irradiation of 2 Gy is applied to donor cells before irradiated cell culture medium is collected and transferred to recipient cells. Data presented here include n values and bystander surviving fraction for the parent HCT116 p53+/+ line and clones B through G. Correlation gives an r^2^ value of 0.44.

Of all clonal lines presented, clones A, F and D did not display a significant decrease in cell survival following addition of ICCM indicating there was no or a weak bystander signal. Figure 5 A and B display the cell surviving fraction of the parent and clone A, however, clone A exposed to direct ionizing radiation shows less cell death (71% surviving faction) compared to that of the parent population (57% surviving fraction). Most clonal lines derived from a non-irradiated parent line showed signficiantly stronger bystander signals (P < .0001). Clonal lines subject to irradiation prior to isolation significantly differed in bystander signal strength in comparison to clonal lines which were not initially irradiated (P = .055). A correlation between *n* value and bystander signal strength was also observed irrespective of whether the clone was derived from irradiated or non-irradiated parent populations (Figure 6). Figure 6 displays the relationship between *n* value and surviving fraction following bystander treatment where clone A is omitted due to the unusually high shoulder size.

## Discussion

Initial findings regarding sublethal damage and defective colonies sparked interest in the idea that cells exposed to x-ray radiation have the potential to form colonies of various sizes.^29,30^ The data presented in this study demonstrate variability that arises in a given population of cells and how radiosensitivities differ across clonal populations. The results show that clonal populations of the same initial culture exhibit variation in radiosensitivity when subject to the same dose. When modeled with the multi-target model, a prominent shoulder region can be observed in clone A suggesting a heightened radioresistant nature compared to the parent population from which it was derived. This prominent shoulder was characteristic of a large *n* value not observed in other clonal populations derived from the same non-irradiated parent population. Most other clonal populations have a relatively smaller *n* value and overall suggest a more radiosensitive nature compared to the previously mentioned clone A.

Both the multi-target and linear-quadratic models were fitted to the data because they are mathematical expressions, which have shown to be good fits to most in vitro data. However, as can be seen here, using parameters from the fittings to compare the shoulder size, especially between the two models, is misleading as the fits are very poor. Therefore, they cannot be used to derive biological mechanistic explanations. However, it is useful to present the results of these fits if only to discount them. Besides, there are several interpretations of the LQ-model apart from Chadwick and Leenhouts derivation with double strand breaks,^31,32^ for example, the ATM-shuttling hypothesis developed by Foray and his group^33,34^ which proposes that delay in ATM-shuttling following radiation exposure causes radiosensitivity.

When all clonal populations were tested for the presence of bystander signals following the bystander medium transfer assay, it was demonstrated that most clonal populations regardless of their origin from a non-irradiated or irradiated population, displayed a significant reduction in cell survival following receipt of ICCM (Figure 4). However, clone A with the largest, and highly unusual, *n* value did not produce bystander signals suggesting a decreasing bystander signal strength with large shoulder size (Figure 4B). Unfortunately, the rest of the clones had n values quite close together, but a correlation plot (Figure 5) does suggest a trend for bystander induced survival reduction to correlate with the n value (r^2^ = 0.44). There is a trend for *n* value to correlate with bystander induced reduction in survival however other additional factors could be involved. Also, while to our knowledge no previous experiments were set up to examine this relationship, there is anecdotal evidence in the literature that shoulder size and bystander signal strength are related inversely. The paper by Mothersill et al (2002) examined parent cell lines and radiosensitive lines with various DNA repair defects derived from these parents.^35^ Irrespective of the nature of the repair defect, all radiosensitive lines were more radiosensitive than their parent line. Also many radioresistant cell lines such as PC3 do not show bystander associated cell death while radiosensitive lines such as SW48 do show strong bystander effects.^35^

In certain clonal populations, bystander signals were not produced even though they were radiosensitive. Consistent with previous findings this could suggest the presence of a low dose hyperradiosensitivity with increased radioresistance as the dose increased (HRS/IRR) mechanism. In instances of hyperradiosensitivity a generally greater than expected response to radiation is observed. However, various studies have shown that certain cell lines only respond to bystander signals in the lower dose region where HRS is seen.^16,15^

Apart from the findings in relation to RIBE, the data in this paper suggest that the mathematical expressions based on classical target theory predictions do not provide good fits to these results. This is important to note because many of the classical experiments were done using a few cell lines such as CHO or V79 cells. These have high plating efficiencies of the order of 80-90% but limited expression of tissue of origin characteristics. Most modern radiobiology is done using lines which express important parameters related to epithelial cell or tumor function, but which have plating efficiencies below 50%. High plating efficiencies are necessary to derive meaningful target theory based conclusions. This is because of the statistical probability that radiation is the cause of a cell not forming a colony if the PE is high. With a low plating efficiency, the cause of not forming a colony need not be the radiation effect. Nowadays the focus is on molecular effects so that plating efficiency is not such an issue.

In conclusion, the data presented show marked clonal heterogeneity in cell lines derived from both irradiated and unirradiated progenitors. This manifests as differences in doubling time, plating efficiency and radiosensitivity. The data also reveal a weak correlation between shoulder size (a surrogate for low dose radioresistence) and the ability of ICCM to reduce the plating efficiency of unirradiated cells. The data also suggest that the commonly used mathematical expressions traditionally used to fit survival curve data provide poor fits to the data in this paper, possibly due to the low plating efficiency of the cell lines. In conclusion, the results may have implications for tumor radiotherapy where clonal heterogeneity is an important limitation for treatment.
